# Leveraging neural dynamics to extend functional lifetime of brain-machine interfaces

**DOI:** 10.1038/s41598-017-06029-x

**Published:** 2017-08-07

**Authors:** Jonathan C. Kao, Stephen I. Ryu, Krishna V. Shenoy

**Affiliations:** 1 Department of Electrical Engineering, University of California Los Angeles, Los Angeles, CA 90095 USA; 2 Department of Electrical Engineering, Stanford University, Stanford, CA 94305 USA; 3 Department of Neurobiology, Stanford University, Stanford, CA 94305 USA; 4 Department of Bioengineering, Stanford University, Stanford, CA 94305 USA; 5 Neurosciences Program, Stanford University, Stanford, CA 94305 USA; 6 Howard Hughes Medical Institute, Stanford University, Stanford, CA 94305 USA; 7 Department of Neurosurgery, Palo Alto Medical Foundation, Palo Alto, CA 94301 USA

## Abstract

Intracortical brain-machine interfaces (BMIs) aim to restore lost motor function to people with neurological deficits by decoding neural activity into control signals for guiding prostheses. An important challenge facing BMIs is that, over time, the number of neural signals recorded from implanted multielectrode arrays will decline and result in a concomitant decrease of BMI performance. We sought to extend BMI lifetime by developing an algorithmic technique, implemented entirely in software, to improve performance over state-of-the-art algorithms as the number of recorded neural signals decline. Our approach augments the decoder by incorporating neural population dynamics remembered from an earlier point in the array lifetime. We demonstrate, in closed-loop experiments with two rhesus macaques, that after the loss of approximately 60% of recording electrodes, our approach outperforms state-of-the-art decoders by a factor of 3.2× and 1.7× (corresponding to a 46% and 22% recovery of maximal performance). Further, our results suggest that neural population dynamics in motor cortex are invariant to the number of recorded neurons. By extending functional BMI lifetime, this approach increases the clinical viability of BMIs.

## Introduction

Intracortical brain-machine interfaces (BMIs) record patterns of action potentials from many neurons in motor cortex and translate them, through a decode algorithm, into control signals to guide prosthetic devices such as computer cursors and robotic arms. These neural signals are recorded via chronic multielectrode arrays implanted into areas of the brain associated with movement generation and planning. An important concern regarding microelectrode arrays is their longevity: as the number of recorded neural signals inevitably decreases through time, BMI performance also declines (e.g., refs 1–3). Hence, a central design goal that is critical to BMI clinical viability is to maximize the functional lifetime of the BMI in the face of worsening multielectrode array recordings. While this concern has implications on the functional lifetime of the BMI, we emphasize that chronic electrode arrays, including the Utah electrode array (Blackrock Microsystems) employed in this study, last long enough to be highly appropriate for nonhuman primate research (e.g., refs 4–9) and for use in clinical trials (e.g., refs 10–16). The Utah array, in particular, has been documented to last for months to years^17–19^. Further, we note that other electrode array technologies have also been successfully employed in non-human primate research (e.g., refs 20–25) but are not currently approved for use in clinical trials. Irrespective of the type of microelectrode array used, we sought to extend the functional lifetime of the BMI beyond when it would have normally failed due to the inevitable decline in multielectrode array recording quality. Our approach to achieve this is to augment the decode algorithm, an intervention implemented entirely in software, and is therefore generally applicable to BMI systems regardless of the specific hardware being used.

**Figure 1:**
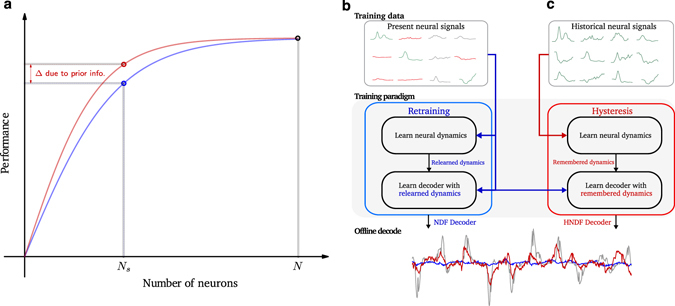
Decoder hysteresis and exploiting neural dynamical invariance. (**a**) Illustration of the decoder hysteresis concept. The blue curve represents the hypothetical drop off in performance in the scenario where neural recording electrodes are lost and a decoder is retrained naïvely with the remaining neurons. The red curve illustrates the idea of using prior information regarding motor cortex, specifically knowledge about neural dynamics, to augment the decode algorithm and mitigate the loss of performance as neurons are lost. (**b**) Block diagram for retraining with remaining neural signals. The present neurons are used to retrain a decode algorithm, which involves neural systems identification and decoder parameter learning to predict kinematics. When decoding withheld testing data to reconstruct a monkey’s hand velocity in the horizontal direction (gray trace), the retrained decoder (blue trace) poorly reconstructs the hand velocities. (**c**) Block diagram for decoder hysteresis. A historical dataset is used to identify neural dynamics of motor cortical activity. These dynamics are remembered and constrain the learning of decoder parameters with the present neural signals. With this approach, the decoded velocity (red) better reconstructs the hand velocities.

Our algorithmic approach capitalizes on prior information, which is readily available soon after the electrode array is initially implanted when many, or even most, electrodes record from one or more neurons. This concept is illustrated in Fig. 1a, where the hypothetical performance of a BMI is plotted as a function of the number of recorded neurons. As time passes and neurons are lost, current practice is to re-learn decoder parameters with the remaining available neurons, a procedure termed “retraining” (Fig. 1a, blue trace). However, a critical oversight with the standard practice of retraining is that it ignores historically-recorded neural data from earlier in the array’s lifetime, when more neurons were available. Although lost neurons are no longer recorded at present, this historical data may nevertheless provide additional information for present decoder training. Specifically, if this historical prior information is invariant to the number of neurons recorded and thus applicable even when few neurons remain, then it may be possible to use this information to beneficially improve decoder parameter estimation. This would amount to using a historical dataset to increase BMI performance at present (Fig. 1a, red trace).

To investigate this concept, we need to determine what this prior information should be and how we can incorporate it into the decoder. We reasoned that two key principles should be embraced. First, this prior information must capture structure in the neural population, so that historically-recorded neurons are informative of the activity of the remaining recorded neurons. Importantly, recent evidence suggests that network structure and correlated behavior in the neural population play an important role in motor control^26–30^ and BMI control^31–33^. This first principle rules out decoders where parameter inference assumes neuron independence, including maximum-likelihood approaches to train velocity Kalman filters^34, 35^ and the state-of-the-art FIT Kalman filter^6, 36^. Because historically-recorded neurons are no longer observed today, the neuron independence assumption implies that once neurons are lost, they are uninformative of the remaining neural signals. The second principle embraced is that this prior information should be invariant to the number of neurons being recorded. Of the millions of neurons participating in any motor behavior, an electrode array merely eavesdrops on tens to hundreds of neurons, which is an extremely small sampling of the involved neurons. This prior information should not capture dramatically different views of underlying structure as a result of sampling. If these principles hold, then it is possible that this prior information can beneficially improve decoder parameter inference even after many neurons have been lost from view.

Based on these principles, we propose that this prior information should reflect neural population dynamics during reaching^7, 30, 37–39^. Neural population dynamics capture network structure by describing how populations of neurons modulate their activity through time in lawful ways to generate motor behaviors. Specifically, neural dynamical rules describe how the neural population activity at time *k* is informative of the population activity at time *k* + 1. Studies have demonstrated that these neural population dynamics exhibit similar structure across many different monkeys and humans^7, 40^, can better explain the heterogeneity and correlated behavior in neural populations than traditional models^26, 30, 41, 42^, are good predictors of motor reaction time^43, 44^, and can be incorporated into BMIs to substantially increase performance^32, 33^. Further work has also demonstrated that similar dynamics arise in recurrent neural networks trained to generate hand kinematics or EMG^30, 38, 39^. A key consequence of hypothesizing motor cortex to be a dynamical system for movement generation is that the neural dynamics, if lawful, should be invariant under the same motor behaviors no matter the quality of experimental neural observations. Although inference of these neural dynamics will vary depending on the quality of neural observations, our estimate of these neural dynamics should be statistically consistent, converging to the underlying neural dynamics as more neural data is available^29^. Based off the finding that modeling neural population dynamics increases BMI performance^32, 33^, we hypothesize that using our “best estimate” of neural dynamics, inferred from an earlier point in the array lifetime when more neurons were observed, should result in superior BMI control at present when fewer neurons are available. We infer neural dynamics using expectation maximization, which finds locally optimal maximum-likelihood neural dynamics. Thus, when few neurons remain, this approach rejects locally optimal maximum-likelihood neural dynamics inferred from a small population of neurons in favor of neural dynamics learned from a larger set of neurons in the past.

We implemented this concept algorithmically to extend BMI functional lifetime. In our implementation, instead of retraining a decoder with the remaining neurons available today (Fig. 1b), we “remember” neural dynamics inferred from historically-recorded neural data, and learn new decoder parameters subject to the constraint that the neural population activity evolves according to the remembered dynamics (Fig. 1c). By doing so, we assume that neurons recorded in the past are informative of the neural dynamics in motor cortex, and that the remaining neurons are described by these dynamics. We found that this approach rescues BMI performance following severe channel loss, thereby extending the BMI functional lifetime. We call this application of neural dynamical invariance to a BMI task “decoder hysteresis,” because neural dynamics from a prior state (historically-recorded data) is used to augment BMI performance at the present state (when fewer neurons remain).

We note that our approach is fundamentally different from other approaches where BMI performance is increased via adaptation of neural responses. The neural adaptation approach improves poorly performing BMIs that are characterized by a mismatch in the neural-to-kinematic mapping, as may arise due to several factors including sub-optimal decoder weights^23, 31, 45, 46^ or unexpected neuron loss^47^. Further, the neural adaptation approach differs from the biomimetic decoder design approach, which seeks to minimize the need for learning and adaptation by building a decoder whose control strategy is similar to that of the native arm^36, 48, 49^. The biomimetic design approach thus takes corresponding observations of neural and kinematic data, and attempts to mimic the native neural-to-kinematic mappings as closely as possible. Importantly, the performance of decoders leveraging neural adaptation to increase performance have not yet been demonstrated to exceed the performance of biomimetic decoders^50^. Further, BMI users do not demonstrate substantial neural adaptation^36^ or performance improvements through time associated with learning when using biomimetic decoders across days^51^. As we sought to maximize usable BMI performance in the scenario where one knows what neurons have been lost, we compared performance to the biomimetic approach; concretely, this means that we compared performance to a supervised decoder trained with the remaining neurons rather than training a sub-optimal decoder that then leverages neural adaptation to improve performance. We also note that our approach also differs from a recent study that made decoders robust to future unexpected neural variability by training recurrent neural networks with months-to-years of data^52^. This work utilizes historical datasets to improve decoder robustness by sampling neural variability so that when similar neural variability is encountered in the future, it is better able to decode such activity. When few neurons remain, these approaches do not incorporate any historical prior information in a different way to increase performance.

Finally, in designing this study, we compared the performance of our approach to two state-of-the-art biomimetic decoders at the time of our study because we sought to demonstrate an improvement over highly performing decoders in the literature. First, we chose to compare performance to the neural dynamical filter (NDF)^32^, which has been demonstrated in direct closed-loop experiments to outperform the optimal linear estimator^12, 20, 22, 53, 54^, the position-velocity Kalman filter^11, 55^, and the Wiener filter^5, 10, 23, 56^. This comparison also allows us to evaluate whether it is better to re-learn new dynamics for a given subset of neural signals (NDF) or remember dynamics from a historical dataset (decoder hysteresis). We also compared performance to a state-of-the-art decoder incorporating a kinematic dynamical model, the feedback-intention trained Kalman filter (FIT-KF)^36^. The FIT-KF is a variant of the ReFIT-KF, which increased performance over the velocity Kalman filter by a factor of approximately 2×^6^. A decoder outperforming the NDF and FIT-KF at low neuron count regimes would, by transitivity, be expected to also outperform population vector decoders, kinematic-state Kalman filters, Wiener filter decoders, and neural adaptation approaches.

## Results

We tested the decoder hysteresis idea by having monkeys perform a BMI task where they controlled a neurally-driven cursor to acquire targets presented in a virtual environment. We recorded neural activity (threshold crossings at −4.5× root-mean-square voltage) from electrode arrays implanted in dorsal premotor cortex (PMd) and primary motor cortex (M1) as the monkey performed a center-out-and-back reaching task (Methods). Monkey J had two 96-electrode arrays, one implanted in caudal PMd and one in gyral M1, while Monkey L had one array implanted at the border of caudal PMd and gyral M1. We then trained and evaluated the performance of decoders in both offline simulations and closed-loop experiments. We call the novel decoder, which remembers neural dynamics from when more neurons were available, the hysteresis neural dynamical filter (HNDF). Training the HNDF involves remembering the matrices describing the dynamical state-update equation of a linear dynamical system, which is further detailed in the Methods. In offline experiments, we used dynamics inferred from all available neural data collected on March 5, 2011 (January 28, 2013) in Monkey J (L) for use in HNDF decoders built between March 3, 2014 to April 9, 2014 (January 28, 2013 to May 31, 2013). To demonstrate a consistency in the neural dynamics across time, we also performed an additional offline experiment using dynamics inferred from data collected on March 4, 2011 for Monkey L. In closed-loop experiments, we used dynamics inferred from data collected on December 11, 2012 (January 28, 2013) for Monkey J (L) for experiments performed between May 18 to 21, 2015 (May 28 to June 4, 2015). These dates were chosen because they correspond to among the earliest dates during which we built and evaluated an NDF in closed-loop control for each monkey. Thus, the inferred dynamics were from datasets recorded at least two years prior to closed-loop experiments. In closed-loop experiments, we compared HNDF performance to two state-of-the-art decoders: (1) the neural dynamical filter (NDF decoder)^32^ and (2) the FIT-Kalman filter (FIT-KF decoder)^36^, which assumes a velocity tuning model^57^.

### Remembering neural dynamics when more neurons are available increases BMI performance

**Figure 2:**
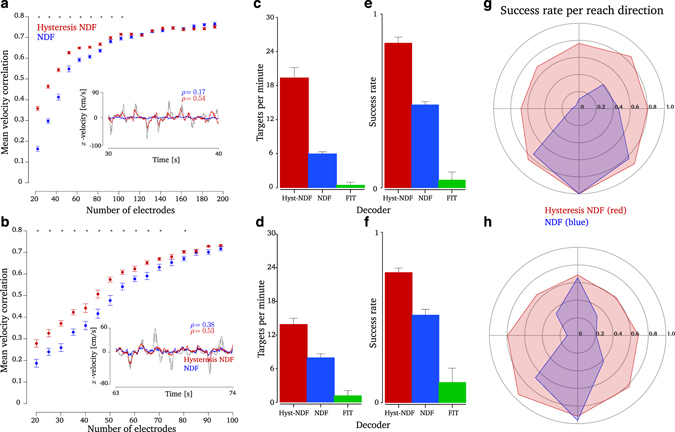
Offline and online evaluation of decoder hysteresis. (**a**) An offline simulation with Monkey J, where electrode loss is simulated and offline decode performance (mean correlation in reconstructing hand velocity) is measured. When 90 or more neural electrodes were lost, the HNDF achieved significantly higher offline decode performance than the NDF (denoted by *p < 0.01, Wilcoxon signed-rank test). The inset shows an example of decoded *x*-velocity (true hand velocity in gray, NDF decoded velocity in blue, and HNDF decoded velocity in red) when 140 electrodes were lost. In this example, the NDF essentially loses a degree-of-freedom of control, being unable to generate velocities in the *x*-direction, while the HNDF rescues the BMI by recovering the lost degree-of-freedom. (**b**) Same as (**a**) but for Monkey L. When 40 or more neural electrodes were lost, the HNDF achieved significantly higher offline decode performance than the NDF (*p* < 0.01, Wilcoxon signed-rank test). The inset shows an example of decoded *x*-velocity when 50 electrodes were lost. (**c**) The performance of the HNDF, NDF, and FIT-KF in closed-loop experiments for Monkey J, with the simulated loss of 110 electrodes. The HNDF performs significantly better (19.4 radial targets per minute) than the NDF (6.0 radial targets per minute) and FIT-KF, which was uncontrollable on 19 out of 20 sessions. Datasets **J_2015-05-18, J_2015-05-19, J_2015-05-20, J_2015-05-21** comprising 3,642 NDF trials and 3,903 HNDF trials. (**d**) Same as (**c**) but for Monkey L, with the simulated loss of 60 electrodes. The HNDF performs significantly better (13.9 radial targets per minute) than the NDF (8.0 radial targets per minute) and the FIT-KF, which was uncontrollable on 14 out of 16 sessions. Datasets **L_2015-05-28, L_2015-05-29, L_2015-06-02, L_2015-06-03, L_2015-06-04** comprising 4,568 NDF trials and 4,646 HNDF trials. (**e**) Success rate of radial target acquisition for Monkey J. The HNDF acquired targets at far higher success rates (83%) than the NDF (48%). (**f**) Same as (**e**) but for Monkey L. The HNDF acquired targets at far higher success rates (76%) than the NDF (52%). (**g**) Target success rate to each center-out target in the workspace averaged over all sessions for Monkey J. There was a deficiency in the NDF to the upper and left parts of the workspace. (**h**) Same as (**g**) but for the Monkey L. There was a deficiency in the NDF along the *x*-axis.

To evaluate if remembering neural dynamics can help mitigate performance loss, we performed an offline simulation of a worst-case neuron loss scenario and evaluated the performance of the NDF vs HNDF decoders. We artificially removed the most informative electrodes based on the mutual information between the electrode’s spiking activity and reach direction (Methods). Monkey J had 192 total electrodes, while Monkey L had 96 total electrodes. We note that although we are strictly simulating electrode loss, the loss of electrodes corresponds to a loss of neurons; for the rest of the manuscript, we will refer to the loss of electrodes as the loss of neurons. On 16 (18) experimental datasets where Monkey J (L) performed a center-out-and-back reaching task with the native arm, we trained an NDF and HNDF decoder with the remaining neurons and evaluated its cross-validation performance in reconstructing the monkey’s hand velocity. We confirmed that, as expected, performance substantially declines as neurons are lost. In contrast and as desired, we found that the HNDF decoder (red) achieved significantly better velocity reconstruction than the NDF decoder (blue) at low neuron counts (more than 90(40) electrodes dropped for Monkey J (L), Wilcoxon signed-rank test, *p* < 0.01). These results are shown in Fig. 2a,b, where the HNDF decoder mitigates performance loss as the number of neurons decreases, showing a similar trend to Fig. 1a. Another common offline metric, mean-square-error in decoded position, also demonstrated better performance on average with the HNDF decoder than the NDF decoder (Supplementary Fig. 1). The same results, using a remembered dynamical system from approximately three years before offline experiments (from March 4, 2011), led to the same qualitative results in Monkey L (Supplementary Fig. 2). Further, we performed an offline analysis comparing the dropoff in performance to the optimal linear estimator^53^ as well as the velocity Kalman filter^58^ (Supplementary Fig. 3). In both cases, we found that the HNDF had a shallower decline in relative performance as the number of neurons decreased. These results demonstrate that, following severe neuron loss in offline simulations, the HNDF decoder is able to decode significantly more accurate velocities from motor cortical spiking activity than a state-of-the-art NDF decoder retrained on the remaining signals. The HNDF decoder achieves this performance improvement by remembering neural dynamics from when more neurons were observed.

Given these offline results, we next asked if the HNDF decoder could increase closed-loop BMI control performance after substantial neuron loss. Evaluating decoders in closed-loop experiments is critical as BMIs are closed-loop feedback systems where the subject can adjust his or her neural activity as a result of visual feedback of the decoder output^59–61^. To this end, we compared the performance of the HNDF and NDF decoders in a closed-loop BMI center-out-and-back cursor control task. We also compared performance to the FIT-KF decoder, which is a state-of-the-art decoder based on velocity-Kalman filtering^6, 36^. We simulated the loss of 110 (60) electrodes in Monkey J (L) to substantially cripple the BMI. We intentionally operated in this difficult-to-control BMI regime so as to best mimic a clinical BMI system on the verge of completely failing, as would be encountered prior to needing to surgically replace a microelectrode array. At this level of electrode loss, offline decode results indicated the HNDF decoder achieved significantly higher performance than the NDF decoder. To keep the monkey engaged in the task, as performance had substantially worsened (Supplementary Movie 1), we made the center-out-and-back task easier by making: the radial targets closer (6 cm away from the center), the acceptance windows larger (width 6 cm), and the hold time shorter (300 ms) (Methods). Even with this easier task, both monkeys were often unable to perform the task with the FIT-KF decoder, failing to control the cursor on 19 of 20 sessions (14 out of 16) in Monkey J (L). We found that both monkeys were able to control the NDF decoder and HNDF decoder to perform the task, and further found that the HNDF decoder achieved substantially higher performance than the NDF decoder. Specifically, while the NDF decoder acquired 6.0 (8.0) radial targets per minute in Monkey J (L), the HNDF acquired 19.4 (13.9) radial targets per minute (Fig. 2c,d, 4 (5) experimental days comprising 7,545 (9,214) trials in Monkey J (L)). At these levels of electrode loss, this corresponds to an increase in the proportion of radial targets acquired by a factor of 3.2× (1.7×). Assuming a peak acquisition rate of 35 targets per minute, typical for modern high-performance systems, the HNDF recovered 46% (22%) of the peak performance. We also observed that the HNDF decoder was able to acquire targets at a higher success rate than the NDF decoder (Fig. 2e,f). Specifically, the HNDF decoder achieved a success rate of 83% (76%) in radial target acquisition, which was significantly higher than that of the NDF decoder 48% (52%) (*p* < 0.01 in both monkeys, Wilcoxon signed-rank test). Of successfully acquired targets, the target acquire time of the HNDF decoder, 1150 ms (1544 ms), was on average faster than that of the NDF decoder, 1314 ms (1627 ms), as shown in Supplementary Fig. 4a,b. This acquire time difference was significant in Monkey J (*p* < 0.01, Wilcoxon signed-rank test) but not in Monkey L. Movies of Monkey J controlling the NDF and HNDF decoders on this task are shown in Supplementary Movies 1 and 2, respectively. Together, these results demonstrate that by remembering neural dynamics from a time when the electrode array afforded measurements from more neurons, BMI control and performance can be substantially increased.

### Remembering neural dynamics can rescue lost degrees-of-freedom of control

An important qualitative observation is that, following severe neuron loss, the HNDF decoder was able to span more of the task workspace than the NDF decoder. The NDF decoder consistently displayed significant anisotropy in velocity control, whereas the HNDF decoder was able to more uniformly generate velocities in all directions. In offline simulations, we found the NDF decoder sometimes lacked the ability to generate velocities in certain directions (Fig. 2a,b, blue traces in inset), essentially losing a degree-of-freedom of control that would incapacitate it in closed-loop settings. On the other hand, we found that the HNDF decoder was capable of generating velocities in directions where the NDF decoder was deficient, essentially recovering the lost degree-of-freedom of control (red traces in the Fig. 2a,b inset). These control anisotropies in the NDF decoder were similarly reflected in closed-loop BMI experiments. Monkey J had difficulty reaching the upper left part of the workspace using the NDF decoder, although he was capable of acquiring all targets with the HNDF decoder (Fig. 2g). Monkey L had difficulty reaching in the horizontal direction with the NDF decoder, but was capable of reaching all targets with the HNDF decoder (Fig. 2h). One way to view these results is in a dynamical systems perspective. In the NDF and HNDF decoders, we are using a dynamical system to infer a *neural population state*, which is a low-dimensional projection of the neural activity that summarizes the correlated activity across the population (see Methods). Critically, each decoder’s neural dynamical model influences the trajectory of the neural population state^32^. Hence, these observations imply that the remembered dynamical model (HNDF decoder) drives the neural state to traverse regions of state-space that are more informative of BMI kinematics. In contrast, the re-learned dynamical model (NDF decoder), while locally optimal in explaining the neural activity in a maximum-likelihood sense, drives the neural population state in subspaces that are less informative of BMI kinematics.

### The HNDF utilizes higher-frequency dynamical modes for decoding

**Figure 3:**
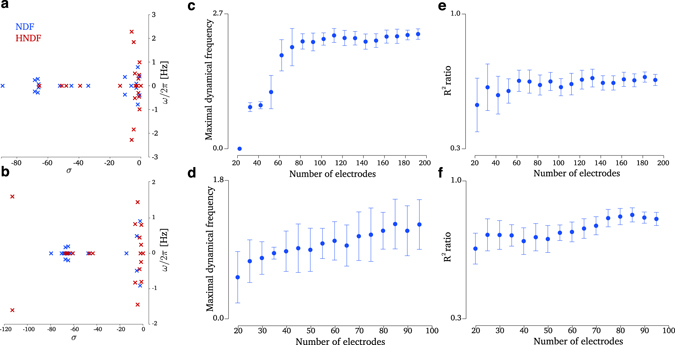
Eigenspectrum and rotational modes. (**a**) Eigenvalue spectrum of the remembered dynamics matrix used for decoder hysteresis (red) and an example eigenvalue spectrum of re-learned neural dynamics (blue) with the simulated loss of 140 electrodes in Monkey J. The frequencies used by the re-learned dynamics are smaller than those of the remembered dynamics. (**b**) Eigenvalue spectrum of the remembered dynamics matrix used for decoder hysteresis (red) and an example eigenvalue spectrum of re-learned neural dynamics (blue) with the simulated loss of 60 electrodes in Monkey L. (**c**) The max frequency of the dynamical system versus the number of electrodes used to infer the dynamics in Monkey J. We only considered oscillation frequencies for modes which had a time constant of decay greater than 20 ms, since the timescale of the exponential decay would be faster than any oscillation. The oscillation frequencies of the neural dynamics decrease as the number of electrodes decreases (linear regression *r* ^2^ = 0.59, slope significantly different than 0 at *p* < 0.01). This trend also held up using the average frequency of the eigenvalues of the dynamical system. (**d**) Same as (**c**) but for Monkey L (linear regression *r* ^2^ = 0.48, slope significantly different than 0 at *p* < 0.01). (**e**) The *R* ^2^ ratio quantifies the ratio in describing the neural population activity with a skew symmetric dynamics matrix (having purely imaginary eigenvalues) vs the least-squares optimal dynamics matrix (having complex eigenvalues) as further described in the Methods. If the *R* ^2^ ratio is large, it signifies that much of the dynamical variance can be captured by a purely rotational dynamics matrix. As electrodes are lost in Monkey J, the *R* ^2^ ratio significantly declines (linear regression *r* ^2^ = 0.13, slope significantly different than 0 at *p* < 0.01), indicating that the neural activity is less well-described by rotational modes. (**f**) Same as (**e**) but for Monkey L (linear regression *r* ^2^ = 0.43, slope significantly different than 0 at *p* < 0.01).

**Figure 4:**
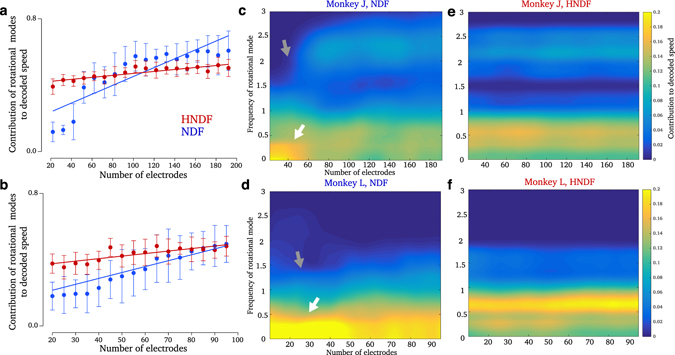
Contribution of rotational modes to decoding. (**a**) Contribution of rotational modes (versus purely exponentially decaying modes) to the decoded speed (Methods) in Monkey J. Error bars denote standard deviation. While both decoders demonstrate a decrease in contribution from rotational modes as electrodes are lost (slopes significantly different than 0, *p* < 0.01), the trend in the HNDF is substantially shallower than in the NDF (difference in slopes, *p* < 0.01). (**b**) Same as (**a**) but for Monkey L. (**c**) Contribution of rotational modes in Monkey J to the NDF, decomposed by frequency of the mode. Contribution from higher frequencies decreases as electrodes are lost (gray arrow) while contribution from lower frequencies and DC modes increases (white arrow). (**d**) Same as (**c**) but for Monkey L. (**e**) Same as (**c**) but for the HNDF decoder. The contributions from rotational modes is more consistent when dynamics are remembered, even as electrodes are lost. (**f**) Same as (**e**) but for Monkey L.

What, then, enables decoders using remembered neural population dynamics to achieve superior performance compared to decoders that re-learn dynamics from fewer available recorded neurons? To address this question, we investigated key differences in the neural dynamics between the HNDF and NDF decoders. We empirically observed that as neuron counts decreased, systems identification with expectation maximization identified neural dynamics with rotational modes having smaller natural frequencies in the NDF (Fig. 3a–d). This phenomenon may result from the neural population activity being more poorly described by rotational dynamics as neuron counts decrease (Fig. 3e,f). Hence, a key difference between the NDF and the HNDF at lower neuron counts is that the HNDF, by remembering a historical neural dynamical model, will drive the neural population state along rotational modes that, on average, have higher natural frequencies. However, do these remembered higher frequency rotational modes meaningfully contribute to the decoded output? For example, it could be that even if higher natural frequencies are present in certain modes, these modes do not contribute to the decoded kinematics at low neuron counts. To evaluate this, we calculated the contribution of all eigenmodes to the decoded output (Methods). We found that, as neuroun counts decreased, rotational modes (i.e., eigenmodes characterized by complex eigenvalues rather than purely real eigenvalues) contributed less to the decoded output in both decoders (negative slopes in Fig. 4a,b, significantly different than 0 at *p* < 0.01). However, the HNDF decoder had a significantly shallower decrease than the NDF (significantly different slopes, *p* < 0.01), indicating that the HNDF utilized a greater contribution from rotational modes to the decoded output at lower neuron counts. When decomposing this contribution by the frequency of the rotational mode, we observed that at lower neuroun counts, the NDF’s decoded output was driven primarily by eigenmodes at DC and low frequencies (Fig. 4c,d, white arrows) and less so by higher frequency rotational modes (Fig. 4c,d, gray arrows). This contribution from DC and low frequencies at low neuron counts from the NDF is qualitatively distinct from how the NDF operates at higher neuron counts, where more data was available for neural dynamical systems identification. In contrast to the NDF decoder, we found that the HNDF decoder maintained a qualitatively similar contribution across all neuroun counts (Fig. 4e,f). That is, following severe neuron loss, the HNDF decoder still utilized the same rotational modes in approximately similar contribution as used when many neurons were available. This consistent contribution across neuron counts was not trivially a result of remembering dynamics, since we allowed the observation process of the dynamical system (and its noise) to be re-learned (Methods). Examples of the velocities decoded by each eigenmode following severe neuron loss are shown in Supplementary Fig. 6. We observe in these examples that the remembered rotational modes of the HNDF supported decoded velocities in directions not accounted for by DC modes (e.g., plane 5 in Monkey J’s HNDF, and plane 3 in Monkey L’s HNDF in Supplementary Fig. 6). Together, these results demonstrate that a key difference in the HNDF is the contribution of rotational modes to decoding, even at low neuron counts, in a fashion consistent with how the NDF operates during high-neuron count BMI use. As the neural dynamics influence the trajectory of the neural state, this observation, coupled with the HNDF achieving better decoding performance than the NDF, suggest that the rotational modes play an important role in driving the neural state along trajectories that aid kinematic decoding.

## Discussion

We demonstrated that, by remembering dynamics learned from an earlier point in an array lifetime, it is possible to increase BMI performance at low neuron counts, extending the functional lifetime of the BMI. This approach relies on the assumption that neural dynamics in PMd and M1 are invariant to the number of neurons being recorded, so that neural dynamics learned when more neurons were available are applicable when few neurons remain. These results therefore suggest that, for a given task, neural dynamics recorded from PMd and M1 are not specific or limited to the exact set of neurons being measured at a given time. If neural population dynamics in a cortical area were specific to the neurons being measured, then the optimal approach to systems identification (and BMI decoder design) would be to re-learn maximum-likelihood dynamics for each specific neural population being recorded. Rather, our results demonstrate that, for decoding kinematics, it is better to instead use the neural population dynamics inferred with as many neurons as possible. Our results are consistent with the hypothesis that, for a given task, there are lawful neural population dynamics that govern the evolution of population neural activity for producing motor behavior. Under this hypothesis, the neural dynamics are statistically consistent, so that they are better inferred as the population size grows larger. We note that an additional analysis, shown in Supplementary Fig. 5, found that in the scenario where the same population of neurons is recorded over time (e.g., as in an optical imaging BMI^62, 63^), remembering the observation process of the dynamical system (Methods), in addition to the dynamics process, resulted in superior offline decoding performance.

We observed that one key difference between the remembered dynamics and the re-learned dynamics was that, at lower neuron counts, the re-learned dynamics did not use higher-frequency rotational modes for decoding. However, the HNDF decoder still reliably decoded using the neural state in these rotational modes in a manner similar to when many neurons remained, suggesting that these dynamical modes play an important role in decoding. These results open the possibility of rescuing decoder performance in scenarios where electrode arrays record few neurons even upon implantation. In these scenarios, when historical data is not available, it may be possible to incorporate a prior that regularizes the neural dynamics to use rotational modes with natural frequencies that are close to those empirically observed in motor cortical activity.

A natural question, given these results, is how many neurons are necessary to infer the underlying neural dynamics? This question is tied to the dimensionality of the neural data and is expected to vary under different task conditions^29^. However, for the purposes of 2D cursor control in our specific experimental setup, our results suggest that as long as approximately 100 electrodes are available, it is possible to reliably infer a dynamical system that achieves relatively good performance in decoding hand velocity (Fig. 2a). These results are reasonable given the observation that the dimensionality of motor cortical activity during 2D reaching spans approximately 10–20 dimensions^7, 26, 64^. Further, with training dataset sizes of 500 trials, lasting approximately 500 s, our result that 100 neural electrodes are enough to reliably infer the dynamics of 2D reaching are consistent with a neural theory of dimensionality and dynamics^29^. As we used neural dynamics inferred approximately two and a half years before the experiments, this suggests that the neural dynamics for our 2D reaching task are fairly stationary through time. This is further supported by an offline analysis of decode performance with dynamical systems remembered from different points in time, going back 3 years in Monkey J and 2 years in Monkey L (Supplementary Fig 7). In addition to being stationary through time, other studies have demonstrated evidence that neural dynamics are similar across several different monkeys^7^ as well as humans^40^.

However, neural dynamics are likely to differ from task-to-task. We consider two examples here. First, in scenarios where the BMI is not controlled in a biomimetic fashion, the BMI user may engage “neural adaptation” to increase BMI performance^23, 24, 46, 50^. Importantly, neural population activity is observed to change during the learning process^31, 65^. Given that neural population activity changes during BMI learning, it is likely that the neural population dynamics also change to support this adaptation. Second, neural populations have empirically been observed to increase in dimensionality as tasks become more complex^29^. Hence, it is likely that in more difficult BMI tasks, such as controlling a multi-degree of freedom robotic arm, the dimensionality of the neural population activity will increase. As neural populations explore new dimensions, the neural dynamics underlying this activity may potentially increase in complexity. In these scenarios, performance may drop off more rapidly with electrode loss, and so remembering the complex dynamics may be especially important in mitigating performance loss. Further, our results suggest that remembering neural dynamics may also be able to rescue lost degrees-of-freedom of control (Fig. 2a,b, insets, and Fig. 2g,h). It is also important to note that this approach relies on having sampled the dynamics of the task before neuron loss. If the BMI task is altered following neuron loss, and the dynamics of the new BMI task are substantially different than in prior tasks used, the hysteresis approach may not generalize. Therefore, it may be beneficial to record during a diversity of relevant and complex clinical tasks soon after array implantation to sample neural dynamics in each of these tasks.

A further observation was that the NDF performed better than the FIT-KF at lower neuron counts. Although prior studies have not directly compared the performance of the NDF and FIT-KF, both decoders achieve comparable bitrates on the grid task, using the same monkeys and the same arrays^32, 36, 66^. Thus, it appears that the performance drop-off as neurons are lost is different for both decoders. This is further supported by the offline simulation in Supplementary Fig. 3, whereby the velocity Kalman filter is shown to degrade in performance at a faster rate in Monkey J. Investigating the key factors in closed-loop control that account for this difference may shed insight into how to further mitigate performance loss. We additionally note that our approach differs from decoders leveraging neural adaptation, where performance can be improved through time as the monkey adapts the firing rate statistics of neurons that control the decoded output^23, 24, 46, 67^. However, these neural adaptation techniques are most appropriate when the decoder is not biomimetic, and have not been demonstrated to exceed the performance of biomimetic decoding. Nevertheless, it may be possible, in the scenario where biomimetic performance is especially poor, that decoder design and neural adaptation may be combined to result in even higher performance^50^. Understanding how neural adaptation may augment biomimetic decoding performance may further rescue performance under neuron loss.

Because our technique is implemented entirely in software, it can be combined with other multielectrode array technologies (aside from the Utah array). Further, we note that the lifetimes of these technologies may be highly variable. For example, in a study with 62 implanted Utah electrode arrays, 56% of arrays had no recordable action potentials within the first year of implantation, while 11% of arrays lasted longer than the approximately two-year long duration of the study^17^, consistent with studies demonstrating usable BMI performance for years^18, 19, 51^. As long as a sufficient number of neurons remain, our technique would increase BMI performance over current decoding approaches. Thus, for failure modes where enough information persists (i.e., non-catastrophic failure), our technique effectively extends the usable lifetime of the array beyond when it would have normally failed. Moreover, this algorithmic technique may be combined with other approaches that are aimed at extending the usable lifetime of a BMI. For example, it should be possible to combine our approach with local field potential decoding when action potentials are no longer recorded on electrodes^2, 3, 68–70^. It will be important to assess the extent to which these complementary approaches may further increase the usable lifetime of electrode arrays. Further, while we demonstrated these results using linear dynamical systems, the dynamics underlying motor behaviors for BMI may be nonlinear (e.g., ref. 71). Therefore, it may be possible that techniques for nonlinear systems identification (e.g., refs 71–73) would not only increase decoder performance^32^, but may also strengthen the decoder hysteresis effect. Nevertheless, even in the linear regime, we have shown that it is possible to extend the usable lifetime of the BMI through software interventions at the algorithmic level. In particular, at the performance levels reported in this manuscript where state-of-the-art decoders failed, it would have been possible for human participants to use the HNDF to type on a radial keyboard^74, 75^. Thus, this approach increases BMI functional lifetime, thereby increasing BMI clinical viability.

## Methods

### Electrophysiology and experimental setup

All surgical and animal care procedures were performed in accordance with National Institutes of Health guidelines and were approved by the Stanford University Institutional Animal Care and Use Committee. All experiments reported were conducted with adult male rhesus macaques (J & L) implanted with 96-electrode Utah arrays (Blackrock Microsystems Inc., Salt Lake City, UT) using standard neurosurgical techniques. Monkey J (L) was 13 (19) years old at the time of experimentation. Electrode arrays were implanted in dorsal premotor cortex (PMd) and primary motor cortex (M1) as visually estimated from local anatomical landmarks. Monkey J had two arrays, one in M1 and one in PMd, while Monkey L had one array implanted on the M1/PMd border.

The monkeys made point-to-point reaches in a 2D plane with a virtual cursor controlled by the contralateral arm or by a brain-machine interface (BMI). The virtual cursor and targets were presented in a 3D environment (MSMS, MDDF, USC, Los Angeles, CA). Hand position data were measured with an infrared reflective bead tracking system (Polaris, Northern Digital, Ontario, Canada). Spike counts were collected by applying a single threshold, set to −4.5× the root-mean-square of the spike voltage per neural electrode. The raw neural observations used for all analyses and closed-loop BMI experiments were binned threshold crossings counted in non-overlapping 15 ms bins. Behavioral control and neural decode were run on separate PCs using Simulink/xPC platform (Mathworks, Natick, MA) with communication latencies of 3 ms. This enabled millisecond timing precision for all computations. Neural data were initially processed by the Cerebus recording system (Blackrock Microsystems Inc., Salt Lake City, UT) and were available to the behavioral control system within 5 ms ± 1 ms. Visual presentation was provided via two LCD monitors with refresh rates at 120 Hz, yielding frame updates of 7 ms ± 4 ms. Two mirrors visually fused the displays into a single three-dimensional percept for the user, creating a Wheatstone stereograph^59^.

All tasks performed in this manuscript were variants of a 2D center-out-and-back task. In all offline analyses as well as when training decoders, each monkey performed a center-out-and-back task where the virtual cursor was controlled with his contralateral arm. In this center-out-and-back task, eight targets are placed with uniform spacing on the circumference of a 12-cm radius circle. In polar coordinates, these eight targets are positioned at 0°, 45°, 90°, and so on. The task begins with prompting a target, positioned at the center of the circle. After successful acquisition of the center target, one of the eight radial targets would be randomly chosen and prompted. After successful acquisition of a radial target, or following the failure to acquire any target, the center target was prompted again. The inter-trial time between successful target acquisition and the next target being prompted was 40 ms. The monkey had to acquire the prompted target by bringing the cursor within a 4 cm × 4 cm acceptance window centered on the target within 2 seconds and hold the cursor within the target acceptance window for 500 contiguous milliseconds. After the successful acquisition of any target, the monkey was given a liquid reward.

When the virtual cursor was controlled by the BMI, a center-out-task with different parameters was used. Because we simulated the loss of many electrodes, following severe array degradation, we had to make the task easier to perform to both keep the monkey engaged in the task and to convey meaningful information through the task. We note that even for these simpler center-out-and-back task parameters, a human capable of performing this task would be able to use a radial keyboard to type^74, 75^. Under BMI control following severe neuron loss, the radial targets were moved closer to the center target, being 6 cm apart. The acceptance window was widened to 6 cm × 6 cm, and the hold time to signal target acquisition was shortened to 300 contiguous milliseconds. The monkeys were given 5 seconds to acquire each target before the trial was failed.

For BMI control, we chose an animal model where the monkey is free to move the contralateral arm^3, 6, 32, 51, 74, 76, 77^. We recognize that a limitation of this model is that proprioceptive feedback is present in the neural activity^78, 79^. However, the major motivation for this animal model is that the neural dynamics we are modeling are related to reach generation and movement. Restraining both arms would constrain the neural activity to evolve along dimensions that do not cause overt movement. As these “output-null” dimensions are orthogonal to the “output-potent” dimensions used for movement generation, the dynamics of output-null activity may differ greatly from output-potent activity^80^. This model is consistent with the observation that a human subject using a BMI would be capable of generating neural activity that lives in output-potent dimensions, although this activity would not cause overt movement due to motor injury. We recognize that future studies should better characterize the dynamics of imagined movements in humans with motor injury.

### Decoder algorithms

#### Neural dynamical filter

The neural dynamical filter (NDF) is described in more detail in our previous report^32^. To train the NDF decoder, we perform systems identification to learn a linear neural dynamical system describing population activity. The NDF uses the neural dynamical system to filter the neural observations. It then decodes kinematics linearly from the neural-dynamically filtered activity. The NDF is capable of achieving state-of-the-art levels of performance on 2D cursor control tasks^32^.

Concretely, the NDF models the neural observations of spikes via an autonomous latent-state linear dynamical system (LDS). In the LDS, the observed neural spike counts on each electrode at a time *k*, given by **y**_*k*_, are interpreted as a noisy observation of a low-dimensional and dynamical neural state, **s**_*k*_. The neural state, **s**_*k*_, is a continuous variable that summarizes the activity of the neural population by capturing the correlated structure in the activity. Each dimension of **s**_*k*_, in the case of the LDS, can be inferred as a linear combination of all the observed neurons. The neural state is also *dynamical*, in the sense that knowledge of **s**_*k−*1_ is informative of what **s**_*k*_ will be. In this work, the **y**_*k*_ are the spike counts on each electrode in non-overlapping 15 ms bins. We chose the neural state to be 20-dimensional as to be sufficiently high enough to capture a substantial proportion of the neural variance during reaching^26^. We modeled the LDS in the linear Gaussian form as:

1 $${{\bf{s}}}_{k}={\bf{M}}{{\bf{s}}}_{k-1}+{{\bf{n}}}_{k}$$

2 $${{\bf{y}}}_{k}={\bf{P}}{{\bf{s}}}_{k}+{{\bf{r}}}_{k}$$

where **n**_*k*_ and **r**_*k*_ are zero-mean Gaussian noise terms with diagonal covariance matrices **N** and **R**.

We refer to equation (1) as the dynamics process and the equation (2) as the observation process. The dynamics process describes how the previous neural state, **s**_*k−*1_, is informative of the current neural state, **s**_*k*_, through the matrix **M**. The observation process describes how the observed neural activity, **y**_*k*_, arises from the low-dimensional neural state, **s**_*k*_. Because the covariance matrix **R** is diagonal, the correlated activity in **y**_*k*_ results exclusively from the neural state, **s**_*k*_. If the parameters **M**, **N**, **P** and **R** are known, then the neural state **s**_*k*_ can be inferred from the prior neural state **s**_*k−*1_ and the newly observed neural activity, **y**_*k*_, with the Kalman filter, which is a minimum mean-square error estimator of a Gaussian LDS. This entails a solution of the form:

3 $${{\bf{s}}}_{k}={\bf{M}}{{\bf{s}}}_{k-1}+{{\bf{K}}}_{k}({{\bf{y}}}_{k}-{\bf{PM}}{{\bf{s}}}_{k-1}),$$

where **K**_*k*_ is called the *Kalman gain*, and the term **y**_*k*_ − **PMs**_*k−*1_ is typically referred to as the *innovation*, or what in the neural activity cannot be explained by the neural state. It is possible to derive a recursion for the Kalman gain, **K**_*k*_, the solution of which is:

4 $${{\bf{K}}}_{k}={({\bf{I}}+({\bf{M}}{{\rm{\Sigma }}}_{k-1}{{\bf{M}}}^{{\rm{T}}}+{\bf{N}}){\bf{P}}{{\bf{R}}}^{-1}{{\bf{P}}}^{{\rm{T}}})}^{-1}({\bf{M}}{\Sigma }_{k-1}{{\bf{M}}}^{{\rm{T}}}){\bf{P}}{{\bf{R}}}^{-1},$$

where Σ_*k−*1_ is the covariance of the estimate **s**_*k−*1_. The derivation of this result can be found in ref. 81. Whenever we performed Kalman filtering to arrive at the neural state, we used the steady-state form of the Kalman filter. We found that the Kalman filter converged to its steady-state form on the order of seconds, so that the two decoders were equivalent after a few seconds.

To infer the parameters **M**, **N**, **P** and **R** from experimental training data, we used expectation maximization (EM), which is a maximum-likelihood approach that seeks to maximize the log-likelihood of having observed the neural activity. EM infers parameters in an unsupervised fashion from the sequence of observed neural activity. The time-series of neural observations {**y**}_*k*=1, 2, …, *K*_ were treated as the observed output of a latent state linear dynamical system (LDS). We did not perform any pre-processing steps on the binned spike counts, **y**_*k*_. Briefly, the E-step involves computing the expected joint-log likelihood of the neural state and the neural observations, which can be deduced from the graph structure of the linear dynamical system:

5 $$\mathrm{log}\,p({{\bf{s}}}_{1,\ldots ,K},{{\bf{y}}}_{1,\ldots ,K})=-\sum _{k=1}^{K}(\frac{1}{2}{({{\bf{y}}}_{k}-{\bf{P}}{{\bf{s}}}_{k})}^{{\rm{T}}}{{\bf{R}}}^{-1}({{\bf{y}}}_{k}-{\bf{P}}{{\bf{s}}}_{k}))-\frac{K}{2}\,\mathrm{log}|{\bf{R}}|$$

6 $$\,-\sum _{k=2}^{K}(\frac{1}{2}{({{\bf{s}}}_{k}-{\bf{M}}{{\bf{s}}}_{k-1})}^{{\rm{T}}}{{\bf{N}}}^{-1}({{\bf{s}}}_{k}-{\bf{M}}{{\bf{s}}}_{k-1}))-\frac{K-1}{2}\,\mathrm{log}|{\bf{N}}|$$

7 $$\,-\frac{1}{2}{({{\bf{s}}}_{1}-{\pi }_{1})}^{{\rm{T}}}{{\bf{S}}}_{1}^{-1}({{\bf{s}}}_{1}-{\pi }_{1})-\frac{1}{2}\,\mathrm{log}|{{\bf{S}}}_{1}|-\frac{K(N+d)}{2}\,\mathrm{log}\,2\pi ,$$

where ${{\bf{s}}}_{1}\sim {\mathscr{N}}({\pi }_{1},{{\bf{S}}}_{1})$ and *N* and *d* are the number of electrodes and the dimensionality of the latent state, respectively. The joint log-likelihood, given all parameters, can be computed via Kalman smoothing. The M-step then involves maximizing the parameters (**M**, **P**, **N**, **R**, *π*_1_, **S**_1_) with respect to the joint log-likelihood. We note that while we computed *π*_1_ and **S**_1_, they were of no practical consequence when running in closed-loop after several seconds. The E-step and M-step alternated to increase the log likelihood of the observed data. More details can be found in the reference by Ghahramani and Hinton^82^. When performing EM, we utilized an approximation in the E-step: we assumed that the Kalman smoothing parameters remained constant after convergence of the estimated state covariance matrix within reasonable tolerance. When not using hysteresis, the EM algorithm was initialized with factor analysis. The initial **P** and **R** were the factor loadings and uniqueness matrix, respectively. We subsequently reduced the dimensionality of the spike count data via factor analysis to arrive at a sequence of low-dimensional neural states. The initial *π*_1_ was the mean of the neural states. The initial **S**_1_ and **N** was the covariance of the neural states. The initial **M** was the maximum-likelihood matrix mapping the neural states inferred via factor analysis forward one time step.

After learning the parameters (**M**, **N**, **P** and **R**) via EM, we decoded a sequence of neural states from the training set neural observation. We thus had a sequence of decoded neural states, **S** = [**s**_1_, **s**_2_, …, **s**_*K*_] and a corresponding sequence of observed training set kinematics, **X** = [**x**_1_, **x**_2_, …, **x**_*K*_], where **x**_*k*_ contains the position and velocity of the hand-controlled cursor at time *k*. We then found the matrix **L**_*s*_ which minimizes the mean squared error, ||**X** − **L**_*s*_[**S**; **1**]||^2^, where **1** refers to a row of 1’s appended to the bottom of **S** to allow for a bias to be learned. After defining **S**_*b*_ = [**S**; **1**], the solution is ${{\bf{L}}}_{s}={\bf{X}}{{\bf{S}}}_{{\rm{b}}}^{{\rm{T}}}{({{\bf{S}}}_{{\rm{b}}}{{\bf{S}}}_{{\rm{b}}}^{{\rm{T}}})}^{-1}$.

Consistent with our prior study using this decoder, the decoded kinematics are the 2D position (${\hat{{\bf{p}}}}_{{\boldsymbol{k}}}$) and 2D velocity (${\hat{{\bf{v}}}}_{{\boldsymbol{k}}}$) of a computer cursor. Given that the decoded position and velocity of the cursor at time *k* were ${\hat{{\bf{p}}}}_{{\boldsymbol{k}}}$ and ${\hat{{\bf{v}}}}_{{\boldsymbol{k}}}$ respectively, the decoded position shown to the subject, **p**_*k*_, was calculated as:

8 $${{\bf{p}}}_{k}=(1-\alpha ){\hat{{\bf{p}}}}_{k}+\alpha ({{\bf{p}}}_{k-1}+{\hat{{\bf{v}}}}_{k-1}{\rm{\Delta }}t)$$

with *α* = 0.975 and Δ*t* being the bin width of the decoder. This indicates that the final decoded position is a weighted sum, with 2.5% contribution from the decoded position, and 97.5% contribution from the integrated velocity. The small position contribution in part stabilizes the position of the decoder in the workspace^32, 76^. Other work has noted the importance of taking into account the position contribution of the signal^6^.

#### Hysteresis and memory neural dynamical filter

The hysteresis neural dynamical filter (HNDF) is a variant of the NDF decoder. It utilizes a similar training approach, with a key fundamental difference: with the HNDF, a dataset from an earlier point in the array lifetime is used to infer the dynamics process of the LDS. Concretely, this involves accessing a historically recorded dataset with neural observations $\tilde{{\bf{Y}}}=[{\tilde{{\bf{y}}}}_{1},{\tilde{{\bf{y}}}}_{2},\ldots ,{\tilde{{\bf{y}}}}_{K}]$. Note that we do not require the kinematic information (i.e., **x**_*k*_) from the historical dataset. We then perform EM to infer parameters (**M** ^hyst^, **N** ^hyst^, **P** ^hyst^, **R** ^hyst^) from the neural data $\tilde{{\bf{Y}}}$.

In the HNDF, we remember the parameters of the dynamics process, which are (**M** ^hyst^, **N** ^hyst^). With neural observations recorded today, **Y** = [**y**_1_, **y**_2_, …, **y**_*K*_], we perform a constrained EM algorithm, where we fix **M** = **M** ^hyst^ and **N** = **N** ^hyst^. In this fashion, the dynamics process is constrained to be identical to the dynamics process inferred from the historical dataset. The constrained EM differs in that (1) **M** is initialized to **M** ^hyst^, (2) **N** is initialized to **N** ^hyst^, and (3) in the M-step, we only update parameters for (**P**, **R**, *π*_1_, **S**_1_). After performing EM, we arrive at a new dynamical system, (**M** ^hyst^, **N** ^hyst^, **P**, **R**), which we use to decode a sequence of neural states, ${{\bf{S}}}^{{\rm{hyst}}}=[{{\bf{s}}}_{1}^{{\rm{hyst}}},{{\bf{s}}}_{2}^{{\rm{hyst}}},\ldots ,{{\bf{s}}}_{K}^{{\rm{hyst}}}]$. This sequence of neural states are then used along with the kinematics to infer the mapping **L**_*s*_ in the same way as in the NDF.

For Monkey J’s offline simulations, we used dynamics inferred from data collected on March 5, 2011 for use in HNDF decoders built between March 3, 2014, to April 9, 2014. For Monkey L’s offline simulations, we used dynamics inferred from data collected on both January 28, 2013, as well as March 4, 2011, for use in HNDF decoders built between January 28, 2013 to May 31, 2013. The results of the HNDF simulations using dynamics inferred from all the neural data on January 28, 2013, are shown in Fig. 2, while the HNDF using dynamics inferred using all the neural data on March 4, 2011, are shown in Supplementary Fig. 2. For Monkey J’s closed-loop experiments, we used dynamics inferred from data collected on December 11, 2012, for experiments performed between May 18 to 21, 2015. For Monkey L’s closed-loop experiments, we used dynamics inferred from data collected on January 28, 2013, for experiments performed between May 28 to June 4, 2015.

The memory neural dynamical filter (MNDF) uses an approach similar to the hysteresis neural dynamical filter. The MNDF is used in scenarios when the identity of the observations is the same throughout time. That is, even though neurons will be lost, the remaining neurons were recorded historically, and their identities are known. In these scenarios, it is also possible to remember the observation process from the past, (**P** ^hyst^, **R** ^hyst^). Therefore, the MNDF uses the historically inferred dynamical system, (**M** ^hyst^, **N** ^hyst^, **P** ^hyst^, **R** ^hyst^) to decode a sequence of neural states. Thus, the MNDF does not require an additional EM optimization. After inferring a sequence of neural states, a new **L**_*s*_ matrix is learned for the remaining electrodes in the same way as in the NDF. We note that the MNDF approach is in general implausible for multielectrode data, since over time it is not possible to ensure that the same neurons are measured on each electrode.

#### Feedback-intention trained Kalman filter

The state-of-the-art feedback-intention trained Kalman filter (FIT-KF) is a variant of the recalibrated feedback-intention trained Kalman filter (ReFIT-KF)^6, 36^. The main difference between the FIT-KF and the ReFIT-KF is that the FIT-KF is trained from a reaching dataset, whereas the ReFIT-KF is trained from a dataset under BMI control. We demonstrated that the FIT-KF can achieve the same level of performance as the ReFIT-KF without requiring the collection of an additional BMI control dataset^36^. The major innovation of the FIT-KF relates to an intention estimation intervention performed on the kinematics^36^. Specifically, it is assumed that at every point in the trial, the monkey intends to reach directly to the target, even while his native arm may make a curved reach. Further, it is assumed that once the monkey is within the target acceptance window, he intends to command a zero velocity, even though there may be residual movement in the acceptance window. These assumptions cause the training set kinematics to be altered. Specifically, all velocities during the course of a reach are rotated so that they point directly to the prompted target, and all velocities in the acceptance window of the target are set to zero^6, 36^. We denote these altered kinematics at time *k* as ${\tilde{{\bf{x}}}}_{k}$. We note that, as in ref. 36, the FIT-KF kinematics incorporate the *x*− and *y*− positions and velocities of the cursor, as well as a bias term.

The FIT-KF is a kinematic-state Kalman filter^6, 34–36^ with the following underlying dynamical system:

9 $${\tilde{{\bf{x}}}}_{k}={\bf{A}}{\tilde{{\bf{x}}}}_{k-1}+{{\bf{w}}}_{k}$$

10 $${{\bf{y}}}_{k}={\bf{C}}{\tilde{{\bf{x}}}}_{k}+{{\bf{q}}}_{k},$$

where **w**_*k*_ and **q**_*k*_ are zero-mean Gaussian noise terms with covariance matrices **W** and **Q**. It is worth noting that the **A** and **W** matrices here only model the evolution of the kinematics, and do not capture any information about the neural population activity. The matrices (**A**, **W**, **C**, **Q**) are fit by maximum-likelihood approaches. Given $\tilde{{\bf{X}}}=[{\tilde{{\bf{x}}}}_{1},{\tilde{{\bf{x}}}}_{2},\ldots ,{\tilde{{\bf{x}}}}_{K}]$ and **Y**, it can be shown that:

11 $${\bf{A}}={\tilde{{\bf{X}}}}_{:,2:{\rm{end}}}{\tilde{{\bf{X}}}}_{:,1:\mathrm{end}-1}^{{\rm{T}}}{({\tilde{{\bf{X}}}}_{:,1:\mathrm{end}-1}{\tilde{{\bf{X}}}}_{:,1:\mathrm{end}-1}^{{\rm{T}}})}^{-1}\,$$

12 $${\bf{W}}=\frac{1}{K-1}({\tilde{{\bf{X}}}}_{:,2:{\rm{end}}}-{\bf{A}}{\tilde{{\bf{X}}}}_{:,1:\mathrm{end}-1}){({\tilde{{\bf{X}}}}_{:,2:{\rm{end}}}-{\bf{A}}{\tilde{{\bf{X}}}}_{:,1:\mathrm{end}-1})}^{{\rm{T}}}$$

13 $${\bf{C}}=\,{\bf{Y}}{\tilde{{\bf{X}}}}^{{\rm{T}}}{(\tilde{{\bf{X}}}{\tilde{{\bf{X}}}}^{T})}^{-1}\,$$

14 $${\bf{Q}}=\frac{1}{K}({\bf{Y}}-C\tilde{{\bf{X}}}){({\bf{Y}}-C\tilde{{\bf{X}}})}^{{\rm{T}}},\,$$

where the matrix ${\tilde{{\bf{X}}}}_{:,{\rm{a}}:{\rm{b}}}$ refers taking columns *a* to *b* of the matrix $\tilde{{\bf{X}}}$.

#### Optimal linear estimator

The optimal linear estimator^53^ (OLE) was fit by solving the least-squares regression problem between the sequence of observed kinematics in the training set, **X**, and the corresponding sequence of observed neural data, **Y** = [**y**_1_, **y**_2_, …, **y**_*K*_]. Analogous to the NDF case, we solved for the matrix **L**_*y*_ minimizing the mean squared error ||**X** − **L**_*y*_[**Y**; **1**]||^2^. We then defined **Y**_b_ = [**Y**; **1**], so that a row of 1’s was appended to the bottom of the matrix to account for a bias term. The solution is ${{\bf{L}}}_{y}={\bf{X}}{{\bf{Y}}}_{{\rm{b}}}^{{\rm{T}}}{({{\bf{Y}}}_{{\rm{b}}}{{\bf{Y}}}_{{\rm{b}}}^{{\rm{T}}})}^{-1}$. As pre-processing on the neural data, **Y**, we convolved the activity of each channel with a causal Gaussian kernel having standard deviations 100 ms.

### Mutual information for electrode dropping

When performing electrode dropping experiments, we dropped electrodes according to the mutual information between each electrode’s spiking distribution and the prompted target (i.e., reach direction). For a given electrode, we define the following probabilities.

- *p*_*Y*_(*y*): the probability of observing *y* spikes in a 15 ms window.
- *p*_*X*_(*x*_*i*_): the probability of target *x*_*i*_ being prompted on a given trial.
- *p*_*Y*|*X*_(*y*|*x*_*i*_): the probability of observing *y* spikes in a 15 ms window when the monkey is reaching to target *x*_*i*_.

In addition to this, we let ${\mathscr{Y}}$ denote the set of values *y* can take on, which for our experiments was the set {0, 1, 2, 3, 4, 5+}. The element 5+ indicates instances where 5 or more spikes occurred in the 15 ms window. We also define *N*_*x*_ to be the number of targets. Then, the mutual information between the electrode’s spiking distribution and the prompted target is:

15 $$I(X,Y)=H(Y)-H(Y|X),$$

where

16 $$H(Y)=-\sum _{y\in {\mathscr{Y}}}{p}_{Y}(y)\mathrm{log}\,{p}_{Y}(y)$$

17 $$H(Y|X)=-\sum _{i=1}^{{N}_{x}}{p}_{X}({x}_{i})\sum _{y\in {\mathscr{Y}}}{p}_{Y|X}(y|{x}_{i})\mathrm{log}\,{p}_{Y|X}(y|{x}_{i})\mathrm{.}$$

We calculated the mutual information for each electrode separately. We then ranked electrodes in terms of their mutual information to reach direction. We dropped the most informative electrodes first to simulate a scenario where valuable electrodes were lost early.

### Offline decoding and analysis

The goal of offline decoding is to use a decoder to predict the monkey’s hand velocity from corresponding neural activity. Offline decoding performance is not a reliable predictor of closed-loop performance, where the monkey receives visual feedback of the decoder’s output and can alter his motor response^59–61^. However, it demonstrates the ability of the decoder to reproduce kinematics from the neural activity that generated the observed movements.

For all offline decoding experiments, we used datasets of approximately 500 trials where the monkey performed a center-out-and-back reaching task with the native arm. Of these datasets, 80% of contiguous trials were used for decoder training and the remaining 20% of trials were reserved as held-out testing data.

We measured two metrics when quantifying offline decoder performance: (1) velocity correlation and (2) mean-square error in position. The velocity correlation was calculated as the Pearson correlation coefficient between the recorded hand velocity during reaching and the decoded hand velocity. We calculated the Pearson correlation coefficient separately for the *x*- and *y*-velocities and reported the average of these correlation coefficients. We evaluated the velocity correlations at fixed temporal offsets (or lags) and chose the maximal velocity correlation. The evaluated temporal lags ranged from 15 ms to 90 ms in 15 ms increments. The mean-square error (MSE) was calculated as the average of the norm of the position decode error (defined as the vector from the true hand position to the decoded hand position). As in the velocity correlation, we evaluated the MSE at fixed temporal lags and chose the lag that minimized MSE. To not accumulate position error as a result of previous trials, we reset the decoded cursor position to the hand position at the start of each trial.

### Frequency analysis of neural dynamics

#### Evaluating the eigenvalue spectrum and maximal frequencies

To generate the eigenvalue spectrum shown in Supplementary Fig. 6a,b, we performed an eigenvalue decomposition on the matrix

18 $$\tilde{{\bf{M}}}=\frac{{\bf{M}}-{\bf{I}}}{{\rm{\Delta }}t},$$

where Δ*t* is the bin width used for the decoder (in this work, 15 ms). The matrix, $\tilde{{\bf{M}}}$ represents the first-order approximation of the dynamics process

19 $${\dot{{\bf{s}}}}_{k}=\tilde{{\bf{M}}}{{\bf{s}}}_{k}+{\rm{noise}}.$$

Note that here, ${\dot{{\bf{s}}}}_{k}$ is defined as $({{\bf{s}}}_{k+1}-{{\bf{s}}}_{k})/dt$, i.e., the first-order Euler approximation of velocity. In this fashion, an eigenvalue with a real part 0 indicates that there is no decay along the eigenmode.

The imaginary component of the eigenvalues of $\tilde{{\bf{M}}}$ denote the frequency of each eigenmode. When finding the maximal dynamical frequency used by each dynamics matrix, $\tilde{{\bf{M}}}$, we only considered eigenvalues with time constants greater than or equal to 20 ms. Eigenvalues with smaller time constants would decay so quickly that an oscillation would not persist.

#### Characterization of rotational dynamics via jPCA

To calculate how well the neural population activity could be described by rotational dynamics, we performed jPCA^7^. When performing jPCA^7^, we condition-averaged the neural activity by aligning to the start of a trial. This resulted in the peri-stimulus time histogram (PSTH) for reaches of 16 different conditions (8 center-to-radial conditions, and 8 radial-to-center conditions). Each PSTH was smoothed by convolution with a Gaussian kernel with standard deviation 25 ms and then binned at 15 ms resolution. We performed jPCA by analyzing a robust reaching epoch during the reach (200 ms to 500 ms). We specified 3 jPCA planes, which are a rotation of the top 6 principal components of the neural activity. We calculated the maximal dynamical frequency as the largest frequency used in the matrix **M**_skew_ (see ref. 7). We calculated the *R* ^2^ ratio, describing how well the neural population activity could be described by purely rotational dynamics, as the ratio of *R* ^2^ between the least-squares optimal skew-symmetric dynamics matrix, **M**_skew_ and the least-squares optimal dynamics matrix, **M**_best_. We note that all analyses with jPCA are performed on specific time points on condition-averaged data, where as the dynamical systems found via EM (as in the NDF and HNDF decoders) are inferred from all available single-trial data.

#### Contribution of eigenmodes to decoded output

To calculate the contribution of the dynamical eigenmodes to the decoded output, we decomposed the neural dynamics matrix as:

20 $${\bf{M}}={\bf{U}}{\rm{\Lambda }}{{\bf{U}}}^{-1}\mathrm{.}$$

We then performed a change of basis for the dynamical system by defining ${\tilde{{\bf{s}}}}_{k}={{\bf{U}}}^{-1}{{\bf{s}}}_{k}$. In this manner, the *i* ^*th*^ dimension of ${\tilde{{\bf{s}}}}_{k}$ corresponds to the evolution of the neural state along the *i* ^*th*^ eigenvector with eigenvalue ${{\rm{\Lambda }}}_{i,i}$. The dynamical system under this change of basis is,

21 $${\tilde{{\bf{s}}}}_{k+1}={\rm{\Lambda }}{\tilde{{\bf{s}}}}_{k}+{{\bf{U}}}^{-1}{{\bf{n}}}_{k}$$

22 $${{\bf{y}}}_{k}={\bf{C}}{\bf{U}}{\mathop{{\bf{s}}}\limits^{ \sim }}_{k}+{{\bf{r}}}_{k},$$

and the kinematics are decoded as,

23 $${{\bf{x}}}_{k}={\tilde{{\bf{L}}}}_{s}{\tilde{{\bf{s}}}}_{k}+{{\bf{b}}}_{s},$$

with $${\tilde{{\bf{L}}}}_{s}={{\bf{L}}}_{s}{\bf{U}}.$$

With this dynamical system, we next inferred a sequence of neural states from the neural activity, **S** = [**s**_1_, **s**_2_, …, **s**_*K*_], and then rotated the neural states via $\tilde{{\bf{S}}}={{\bf{U}}}^{-1}{\bf{S}}$. We calculated the contribution of purely decaying eigenmodes (real eigenvalues) or complex planes (paired eigenvalues *σ* ± *jω*) by taking the corresponding modes of ${\tilde{{\bf{s}}}}_{k}$ and decoding velocity. For example, if we wanted to calculate the contribution of a complex plane corresponding to paired eigenvalues *i* and *j*, we would calculate ${{\bf{x}}}_{k}^{i,j}={\tilde{{\bf{L}}}}_{s}^{i,j}[{\tilde{{\bf{s}}}}_{k}^{i};{\tilde{{\bf{s}}}}_{k}^{j}]$, where ${{\bf{L}}}_{s}^{i,j}$ corresponds to the *i* ^*th*^ and *j* ^*th*^ columns of ${\tilde{{\bf{L}}}}_{s}$, $[{\tilde{{\bf{s}}}}_{k}^{i};{\tilde{{\bf{s}}}}_{k}^{j}]$ is the vertical concatenation of the rotated neural states in dimensions *i* and *j*, and ${{\bf{x}}}_{k}^{i,j}$ is a 2-dimensional vector containing the decoded velocities in the horizontal and vertical directions at time *k*. We then calculated the average magnitude of the decoded velocity across all time for this eigenmode, ${r}_{v}^{i,j}={\sum }_{k=1}^{K}\parallel {{\bf{x}}}_{k}^{i,j}\parallel$. The contribution to the decoded velocity of an eigenmode is its contribution, ${r}_{v}^{i,j}$ divided by the sum contribution of all eigenmodes plus a regularization term of 10 cm/s to deal with small overall speeds at low neuron counts.

### Closed-loop performance evaluation

In closed-loop experiments, we primarily evaluated three metrics of performance. They are summarized as follows.

1. **Targets per minute.** Targets per minute denotes, over the course of a 200-trial block, the average acquisition rate of *radial* targets. The acquisition of a radial target involves (a) successfully acquiring the center target and (b) moving the cursor successfully from the center of the workspace to the prompted radial target, and acquiring it by holding within the prompted target’s acceptance window for 300 ms. As such, targets per minute reflects both the accuracy of the decoder as well as the speed of the decoder.
2. **Success rate.** Success rate is the percentage of correctly acquired *radial* targets in a 200-trial block. This metric reflects the ability of the monkey to span the workspace. A higher success rate indicates that the monkey is able to reach more areas in the workspace.
3. **Acquire time.** Acquire time is the time it takes from the target being prompted to when the monkey successfully acquires the target (not including the 300 ms hold time). This metric reflects the speed of the decoder. A decoder with a shorter acquire time is able to move more quickly to the desired target.

To evaluate the performance of online decoders, we had the monkey control all decoders on the same experiment experimental day. We evaluated decoders in an A-B-A-B-A-… fashion, where each letter refers to a decoder. In this fashion, the decoders are repeatedly tested on the center-out-and-back task for 200 trials one after each other. We call each ‘A-B’ segment an experimental block. The experimenter knew the identity of each decoder being evaluated, and all fully completed experimental blocks were included in analysis. The online performance metrics were evaluated for each decoder in each experimental block, and these performance metrics were paired for statistical testing within the same block. We did not use formal effect size calculations to make data sample size decisions, but did perform a variety of experiments with large numbers of decoder comparison trials so as to be able to detect substantial decoder performance differences. To test for a significant difference in each of these metrics, we performed a non-parametric Wilcoxon signed-rank test. The null hypothesis in the Wilcoxon signed-rank test is that the difference in performance amongst the pairs follows a symmetric distribution around zero. Therefore, a significant p-value indicates it is likely that the decoders achieved significantly different performance distributions according to the chosen metric.

### Data availability

Relevant data and analysis code can be made available from the authors upon request.

## Electronic supplementary material

Supplementary Materials

Supplementary Movie 1

Supplementary Movie 2
