# Supplementary material for: Leveraging neural dynamics to extend functional lifetime of brain-machine interfaces
Source: Sci Rep. 2017 Aug 7;7:7395. doi: 10.1038/s41598-017-06029-x (PMC5547077; doi:10.1038/s41598-017-06029-x)
Supplement: Supplementary file 1 — Supplementary Materials [file 41598_2017_6029_MOESM1_ESM.pdf]

Supplementary materials for

Leveraging neural dynamics to extend functional lifetime of brain-machine interfaces

Jonathan C. Kao, Stephen I. Ryu, Krishna V. Shenoy*

*To whom correspondence should be addressed: shenoy@stanford.edu

1 Supplementary Figures

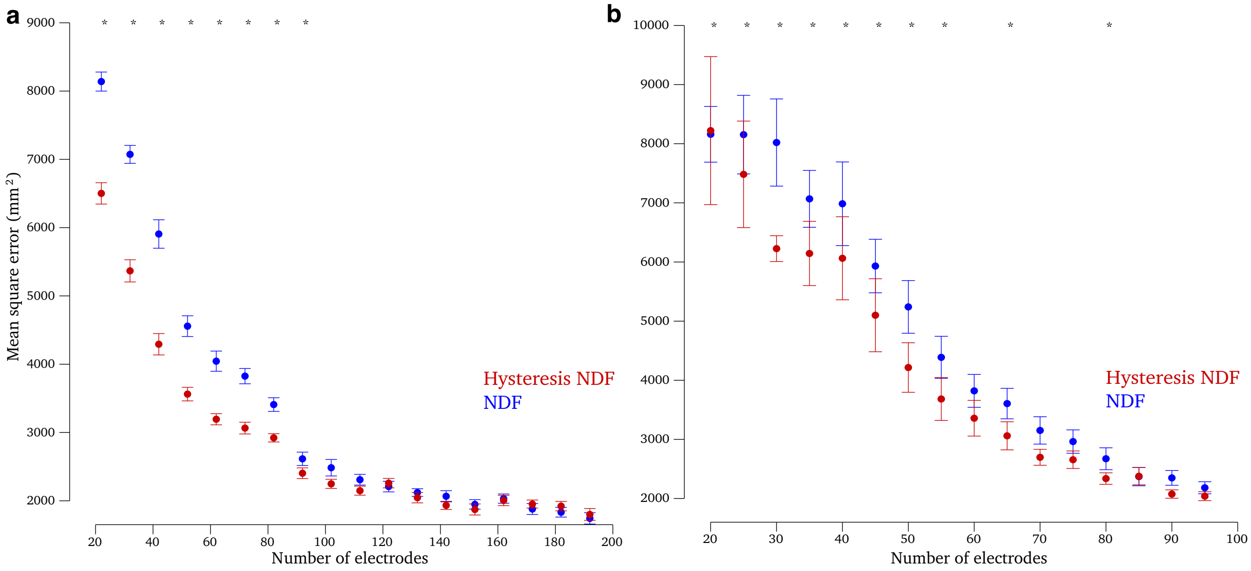

Supplementary Figure 1: Offline simulations for mean-squared error. **(a)** An offline simulation with Monkey J, whereby neural electrode loss is simulated and offline decode performance (mean-square error in hand position) is measured. When 90 or more neural electrodes were lost, the HNDF achieved significantly higher offline decode performance than the NDF ($p < 0.01$, Wilcoxon signed-rank test). **(b)** Same as **(a)** but for Monkey L. Monkey L's mean-squared error was highly variable depending on the experimental day. Following the loss of 40 or more electrodes, the HNDF achieved significantly higher offline decode performance than the NDF ($p < 0.01$, Wilcoxon signed-rank test), except at 50 electrodes lost and more than 70 electrodes lost.

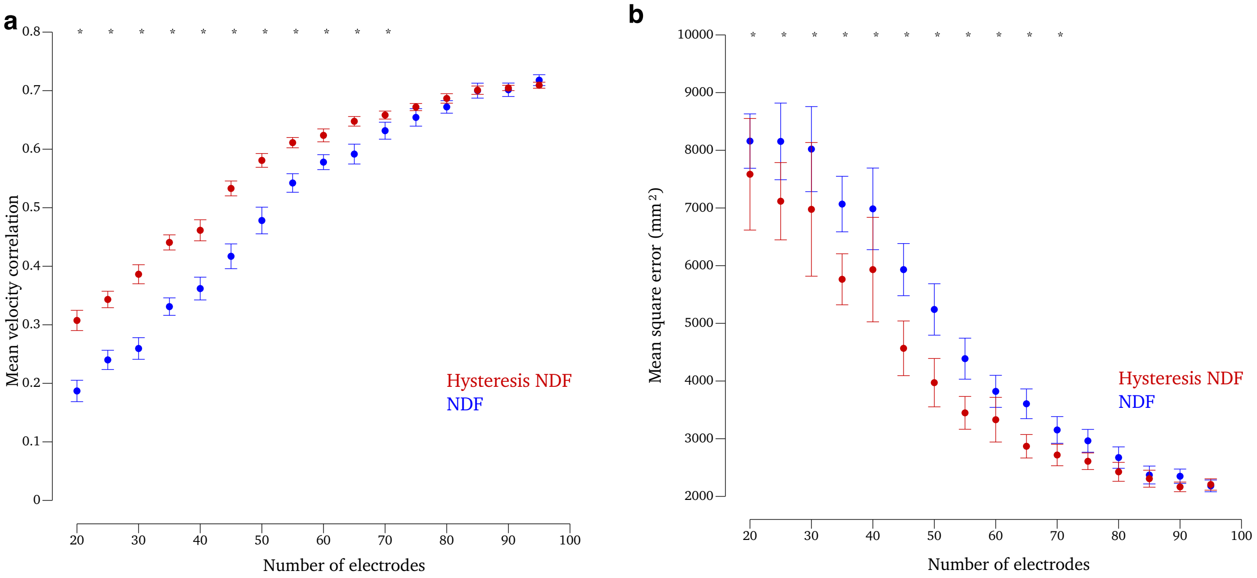

Supplementary Figure 2: Using remembered dynamics from approximately three years ago for Monkey L. These additional offline simulations demonstrate that even when dynamics are remembered from approximately three years before offline experiments in Monkey L (March 4, 2011, see Methods), the same performance trends are observed. **(a)** Here, we simulated electrode loss (as in the main manuscript, see Methods) and report offline decode performance (mean correlation in reconstructing hand velocity) is measured. When 25 or more neural electrodes were lost, the HNDF achieved significantly higher offline decode performance than the NDF (denoted by *, $p < 0.01$, Wilcoxon signed-rank test). **(b)** The same trend held for the mean-squared error, where the HNDF achieved significantly higher offline decode performance than the NDF following the loss of 25 or more electrodes ($p < 0.01$, Wilcoxon signed-rank test).

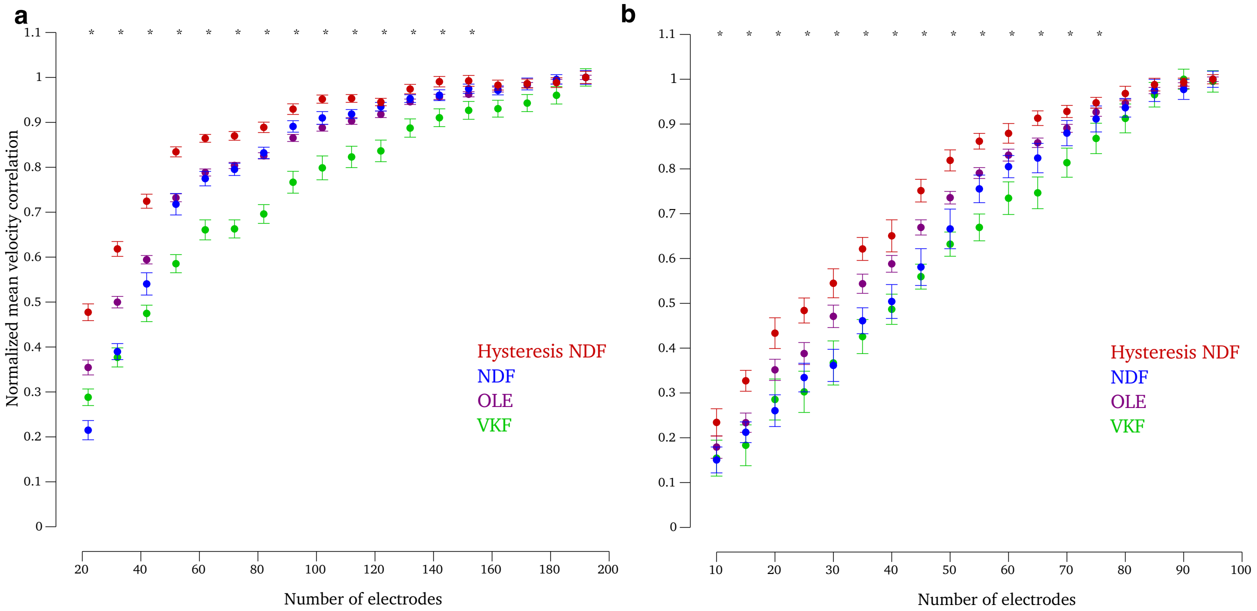

Supplementary Figure 3: Offline simulations of performance loss with the optimal linear estimator (OLE) and velocity Kalman filter (VKF). **(a)** An offline simulation with Monkey J, whereby neural electrode loss is simulated and offline decode performance (relative mean velocity correlation) is measured. Here, we also show the dropoff curves for the OLE and VKF decoders. All velocity correlations are normalized to the peak velocity correlation of that decoder, so that the relative dropoff trends can be seen for each decoder. The *'s denote significant differences in the relative mean velocity correlation between the HNDF and the OLE ($p < 0.01$, Wilcoxon signed-rank test). When evaluating the OLE, we convolved the neural data with a causal 100 ms Gaussian kernel. The mean velocity correlations normalized to were 0.64, 0.83, 0.77, 0.75 for the VKF, OLE, NDF and HNDF, respectively. **(b)** Same as **(a)** but for Monkey L. For the HNDF, we used the remembered dynamics from Supp Fig 2. The mean velocity correlations normalized to were 0.52, 0.76, 0.72, and 0.73 for the VKF, OLE, NDF and HNDF, respectively.

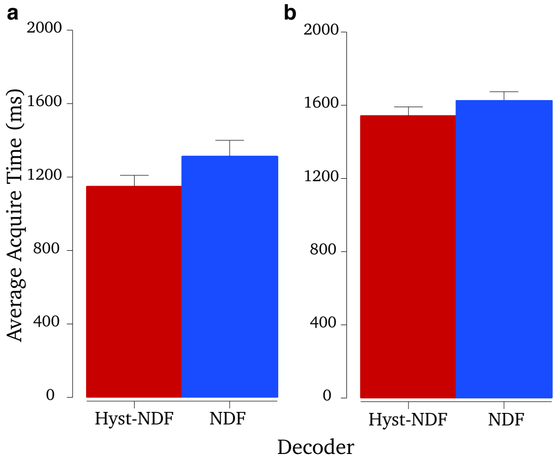

Supplementary Figure 4: Average target acquire time during closed-loop control. **(a)** The average radial target acquisition time per target in closed-loop experiments for Monkey J, with the simulated loss of 110 electrodes. The HNDF average target acquisition time (1150 ms) was on average faster than that of the NDF (1314 ms, $p < 0.01$, Wilcoxon signed-rank test). **(b)** The same as **(a)** but for Monkey L, with the simulated loss of 60 electrodes. The HNDF average target acquisition time (1544 ms) was faster than that of the NDF (1627 ms), although this difference in the means was not significant under the Wilcoxon signed-rank test. This statistic is influenced by the fact that the metric is only measured for successful trials, and while the HNDF is able to acquire more trials successfully, some targets require substantial effort to acquire.

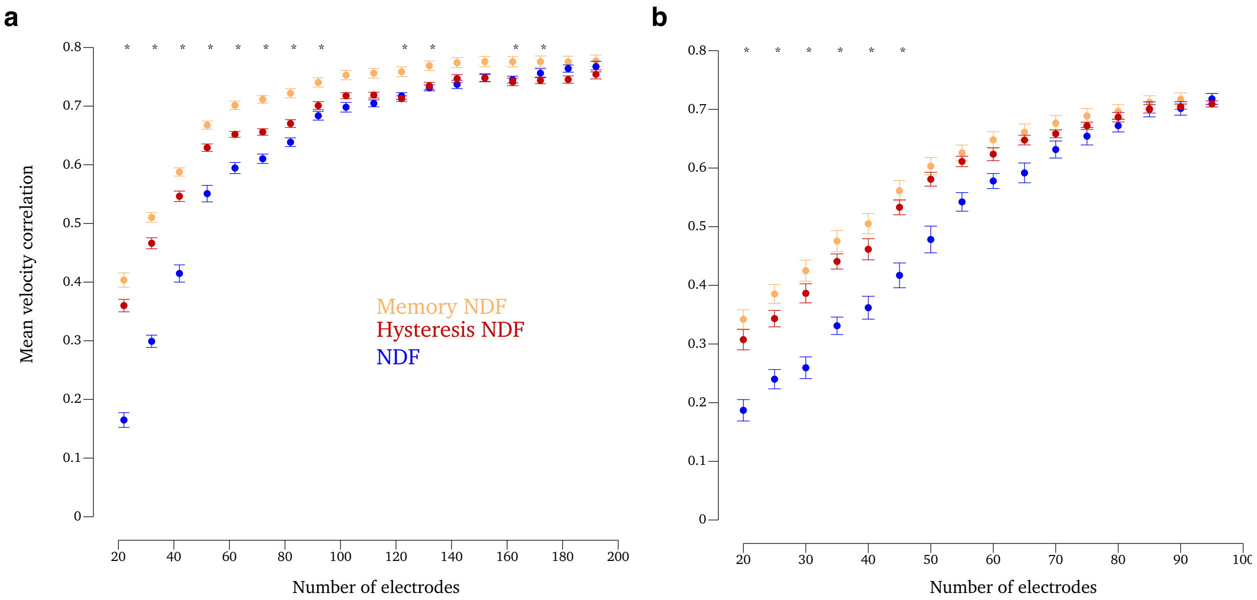

Supplementary Figure 5: Offline performance of the memory NDF (MNDF). **(a)** Offline decode performance as a function of the electrodes lost for a decoder where the entire linear dynamical system is remembered. Remembering both the dynamics and the observation process (orange) increases offline decode performance over remembering only the dynamics process (HNDF, red) and relearning a linear dynamical system (NDF, blue). *'s denote when the MNDF achieved significantly higher performance than the HNDF ($p < 0.01$, Wilcoxon signed-rank test). **(b)** Same as **(a)** but for Monkey L.

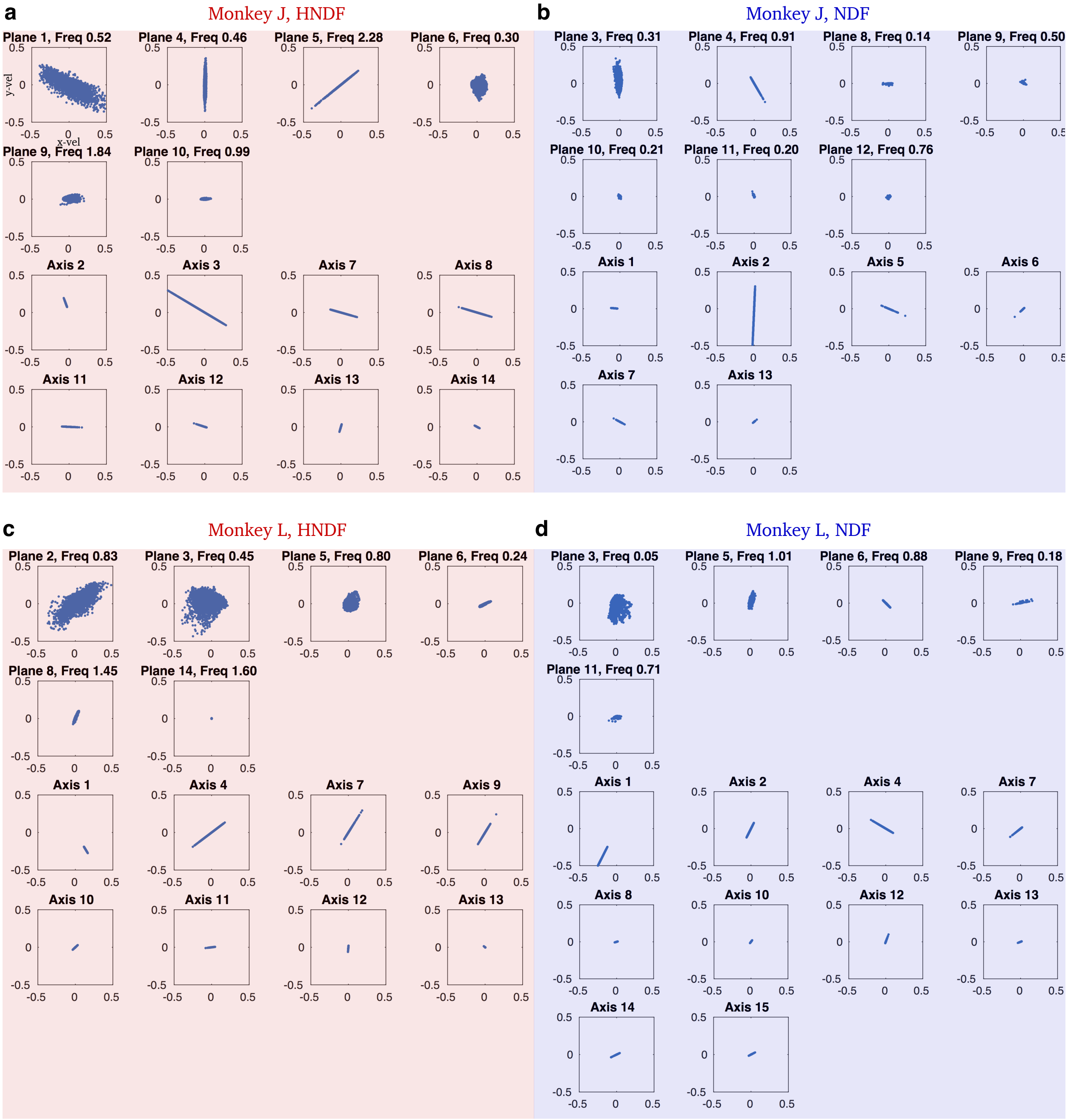

Supplementary Figure 6: Velocities generated by each eigenmode. **(a)** For Monkey J, HNDF decoder with 140 electrodes lost, we show scatter plots of x- and y-velocity (units m/s) generated by a given rotational mode ("Plane") or an exponentially decaying mode ("Axis"). The modes are sorted by their average contribution to the speed of the decoded output. The rotational modes allow for decoding in the up-down direction (Plane 4) as well as along the up-right / down-left directions (Plane 5), which were not decodable from the exponentially decaying modes alone. **(b)** Same as **(a)** but for the NDF decoder. **(c)** Same as **(a)** but for Monkey L with 45 electrodes lost. **(d)** Same as **(b)** but for Monkey L.

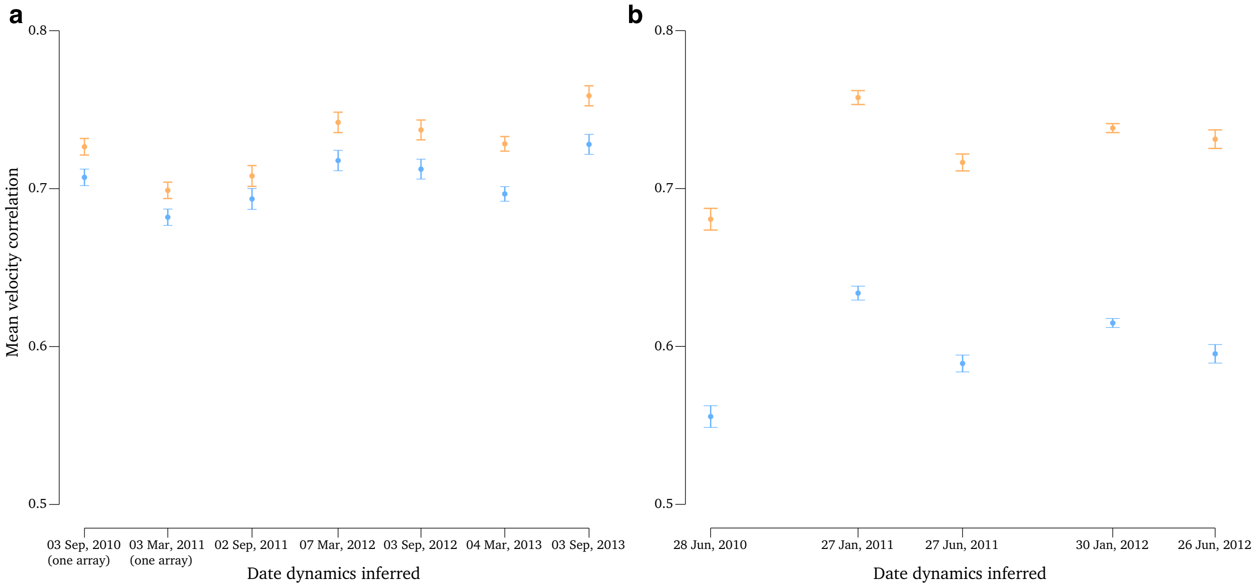

Supplementary Figure 7: Performance for dynamics learned from data in the past. To assess the similarity of dynamics across time, we inferred dynamics matrices from historical datasets and built the HNDF with these historical dynamics. We then evaluated the HNDF performance on datasets used in the offline simulations of Fig 2a,b. We evaluated mean velocity correlation when all electrodes were available (orange) and when half of the electrodes were available (blue). Note that for our data, the Utah arrays have remained relatively stable through time, and thus we are not in the regime where we would expect performance to increase from remembering dynamics in the past. (This is because, with comaprable observations at the present, the dynamics appear to be inferred well.) However, this plot demonstrates that dynamics in the past are applicable today and result in comparable mean performance. This suggests that the dynamics are relatively stable through time with respect to decoding kinematic data. **(a)** For Monkey J, we show the mean velocity correlation when using a dynamics process remembered from the date shown on the x-axis. The dots correspond to the mean performance and the error bars denote the standard error of the mean. The performance remains at a consistent level across the time span of available neural observations. A linear regression for the mean velocity correlation across time when all electrodes were available (orange) had a slope of 2.2×10^{-5}, which is close to zero. Note that the first two points used dynamics inferred from only one array (96 electrodes from Monkey J's M1 array) as during those experiments, we recorded from only one array. **(b)** Same as **(a)** but for Monkey L. Again, the performance remains at a consistent level, even for dynamics inferred in the past. A linear regression for the mean velocity correlation across time when all electrodes were available had a slope of 3.8×10^{-5}, which is also close to zero.

2 Supplementary Movies

Supplementary Movie 1: NDF performance following severe electrode loss. Performance of the NDF decoder (retraining approach) with Monkey J, simulating the loss of 110 electrodes. The NDF decoder is unable to acquire most radial targets, and would therefore be unusable for clinical applications.

Supplementary Movie 2: HNDF performance following severe electrode loss. Performance of the HNDF decoder (hysteresis approach) with Monkey J, simulating the loss of 110 electrodes. The HNDF decoder is able to acquire all radial targets, rescuing BMI performance so that the BMI is now usable.
